# Does Caspase-6 Have a Role in Perinatal Brain Injury?

**DOI:** 10.1159/000375368

**Published:** 2015-03-24

**Authors:** Ana A. Baburamani, Yasuka Miyakuni, Regina Vontell, Veena G. Supramaniam, Pernilla Svedin, Mary Rutherford, Pierre Gressens, Carina Mallard, Satoru Takeda, Claire Thornton, Henrik Hagberg

**Affiliations:** ^a^Perinatal Center, Institute of Neuroscience and Physiology, Department of Obstetrics and Gynecology, Sahlgrenska Academy, University of Gothenburg, Gothenburg, Sweden; ^b^Centre for the Developing Brain, Division of Imaging Sciences and Biomedical Engineering, King's College London, King's Health Partners, St. Thomas’ Hospital, London, UK; ^c^Department of Obstetrics and Gynaecology, Faculty of Medicine, Juntendo University, Tokyo, Japan; ^d^INSERM, U1141, Paris, France; ^e^University Paris Diderot, Sorbonne Paris Cité, UMRS 1141, Paris, France

**Keywords:** Caspase-6, Hypoxia-ischaemia, Q-VD-OPh, Lipopolysaccharide, Microglia

## Abstract

Apoptotic mechanisms are centre stage for the development of injury in the immature brain, and caspases have been shown to play a pivotal role during brain development and in response to injury. The inhibition of caspases using broad-spectrum agents such as Q-VD-OPh is neuroprotective in the immature brain. Caspase-6, an effector caspase, has been widely researched in neurodevelopmental disorders and found to be important following adult stroke, but its function in the neonatal brain has yet to be detailed. Furthermore, caspases may be important in microglial activation; microglia are required for optimal brain development and following injury, and their close involvement during neuronal cell death suggests that apoptotic cues such as caspase activation may be important in microglial activation. Therefore, in this study we aimed to investigate the possible apoptotic and non-apoptotic functions caspase-6 may have in the immature brain in response to hypoxia-ischaemia. We examined whether caspases are involved in microglial activation. We assessed cleaved caspase-6 expression following hypoxia-ischaemia and conducted primary microglial cultures to assess whether the broad-spectrum inhibitor Q-VD-OPh or caspase-6 gene deletion affected lipopolysaccharide (LPS)-mediated microglial activation and phenotype. We observed cleaved caspase-6 expression to be low but present in the cell body and cell processes in both a human case of white matter injury and 72 h following hypoxia-ischaemia in the rat. Gene deletion of caspase-6 did not affect the outcome of brain injury following mild (50 min) or severe (60 min) hypoxia-ischaemia. Interestingly, we did note that cleaved caspase-6 was co-localised with microglia that were not of apoptotic morphology. We observed that mRNA of a number of caspases was modulated by low-dose LPS stimulation of primary microglia. Q-VD-OPh treatment and caspase-6 gene deletion did not affect microglial activation but modified slightly the M2b phenotype response by changing the time course of SOCS3 expression after LPS administration. Our results suggest that the impact of active caspase-6 in the developing brain is subtle, and we believe there are predominantly other caspases (caspase-2, −3, −8, −9) that are essential for the cell death processes in the immature brain.

## Introduction

The immature brain has a high expression of many pro-apoptotic genes in comparison with the adult [for a summary, see [1,2,3]]. Consequently, the activation of apoptotic pathways, particularly caspase activation, following hypoxia-ischaemia has been shown to play a pivotal role in the cell death process [4]. Caspases are cysteine-dependent aspartate proteases that, following activation, regulate apoptosis and inflammation and, more recently, have been reported to have non-apoptotic functions [5]. Caspases can be sub-classified into initiator (caspase-2, −8, −9, −10), effector (caspase-3, −6, −7) and inflammatory (caspase-1, −4 [human], −5, −11 [rodent]). In the developing brain, initiator caspase-2 and effector caspase-3 are abundantly expressed and robustly activated following hypoxia-ischaemia [6,7,8]. Directly targeting specific caspases or broad-spectrum inhibition of caspases using Q-VD-OPh has been shown to be protective in rodent models of hypoxic-ischaemic injury [8,9,10,11,12].

Although classically defined as an effector caspase with homology to caspase-3, studies show that caspase-6 can activate caspase-3 [13], suggesting it may be important in the neonatal brain where caspase-3 is so highly expressed. Recently, Simon et al. [14] reported that caspase-3 can also activate caspase-6 in the process of axonal degeneration, suggesting a dichotomous interaction between these caspases. Whilst pro-caspase-6 expression is low in the fetal and adult human brain [15], caspase-6 has been shown to be crucial in neurodegenerative disorders such as Huntington's, Alzheimer's and Parkinson's disease [16,17], in age-related memory decline [18], following adult stroke [19], and in hippocampal epileptogenesis and epilepsy [20]. From studies in the adult brain, caspase-6 mRNA was upregulated in the ipsilateral cortex within 6 h following focal cerebral ischaemia [21]. Following adult stroke, caspase-6 knockout mice appeared to be protected, with reduced axonal degeneration and an improvement in functional outcome [19]. What function caspase-6 has in the developing brain and its role in immature brain injury remains to be investigated.

Developmentally and following injury, neuronal cell death occurs via apoptosis, and it is microglia, the resident phagocytic cells of the brain, that are involved in both the initiation of cell death and phagocytosing dead and dying neurons [22,23]. This suggests that there could be a tightly regulated interaction between apoptotic cues such as caspase activation and microglial activation. Burguillos et al. [24] have shown that caspase-8 and caspase-3/7 signalling were required for the activation of microglia and, more recently, extracellular caspase-6 was seen to enhance microglial production of TNF-α, regulating inflammatory pain and synaptic plasticity [25]. These studies highlight that greater understanding is required to define the interaction between caspases and microglia.

In this study we investigated the possible apoptotic and non-apoptotic functions caspase-6 may have in the immature brain. First we assessed the spatio-temporal expression and possible contribution of activated (cleaved) caspase-6 following neonatal hypoxia-ischaemia in the development of brain injury. Secondly, we examined the non-apoptotic roles of caspases in inflammatory processes, more specifically, whether the neuroprotective effects that Q-VD-OPh elicits are mediated by modulating microglial activation and to what extent caspase-6 may be involved in microglial activation.

## Materials and Methods

### Human Post-Mortem Case

Informed parental consent was acquired according to the National Health Services UK guidelines, and study ethics were obtained from the National Research Ethics Services (West London), UK (ethics number, 07/H0707/139; Post-Mortem Magnetic Imaging Study of the Developing Brain). The case examined was a pre-term post-mortem brain (gestational age at birth 24 weeks, post-natal age 36 days, post-menstrual age 29.1 weeks) with evidence of white matter injury on post-mortem MRI and histopathology assessment at autopsy.

#### Animals

Mice and rats were bred in-house, kept in a 12-hour light/dark cycle and fed a standard laboratory chow diet and drinking water ad libitum (Experimental Biomedicine, University of Gothenburg, Sweden). All animal experimentation was conducted in accordance with the Department of Agriculture (Jordbruksverket, Sweden) and approved by the Gothenburg animal ethics committee.

##### Genetically Modified Animals

Caspase-6 heterozygote (Het; +/-) mice were purchased from the Jackson Laboratory (cryopreserved stock No. 006236, B6.129S6-*Casp6*^tm1Flv^/J) [26,27]. Caspase-6 Het mice were bred together to get mixed genotypes - caspase-6 knockout (KO; -/-), caspase-6 Het and caspase-6 wild type (WT; +/+). Mixed litters were used for hypoxia-ischaemia experiments.

##### Genotyping

The genotypes of WT, Het and KO mice were determined by polymerase chain reaction (PCR) of genomic DNA extracted from tail clips by a RedExtract-N-AMP Tissue PCR Kit (Sigma-Aldrich, USA). Each PCR reaction (10 μl) contained 1 μl (50-100 ng) of genomic DNA and 5 μl of REDExtract-N-Amp PCR reaction mix, and all three primers were used at a concentration of 1 μm. WT forward primer (CTG AGG GGC GGA GCA CCT TTG CTG, IMR5941), mutant (KO) forward primer (GCC TTC TTG ACG AGT TCT TCT GAG G, IMR5942) and common primer (CTC CAC GGC CTA ATG CAG TTC CTG G, IMR5940) were all ordered from Eurofins MWG GmbH (Ebersberg, Germany). The PCR cycles were 94°C for 3 min, 35 cycles at 94°C for 30 s, 66°C for 30 s, 72°C for 45 s and, finally, 72°C for 2 min. The PCR products were separated on a 1.5% agarose/0.5 × Tris-borate-ethylenediaminetetraacetic acid gel containing SYBR/Safe DNA gel stain (Invitrogen; Life Technologies Sweden). A 100-bp ladder was used to verify PCR products. WT mice were identified with a single band at 620 bp, caspase-6 KO mice with a single band at 340 bp and caspase-6 Het were determined when both bands were present.

#### Induction of Hypoxia-Ischaemia in Neonatal Mice and Rats

Post-natal day 9 (P9) mice were subjected to unilateral hypoxia-ischaemia, essentially according to the Rice-Vannucci model [6,28,29]. This model results in a focal ischaemic injury allowing for a comparison between the injured (ipsilateral) and the non-injured (contralateral) hemisphere. Both female and male pups were used. Briefly, the mice were anaesthetised with isoflurane (4.5% for induction and 2% for maintenance) in a mixture of nitrous oxide and oxygen (1:1), with the duration of anaesthesia being <5 min per pup. The left common carotid artery was isolated and ligated. The pups recovered for 1 h in the parent cage. The litters were then placed in a chamber with a humidified hypoxic gas mixture (10% oxygen in nitrogen, 36°C) for 50 min (mild hypoxia) or 60 min (severe hypoxia). After hypoxic exposure, the pups were returned to their biological dams until the conclusion of the experiment (72 h).

Additionally, P8 Wistar rats were subjected to the same procedure, except the rats were subjected only to 60 min hypoxia (7.7% O_2_). Brains were collected at 8, 24 and 72 h following hypoxia-ischaemia, with control pups not subject to ligation or hypoxia and sacrificed at P8.

#### Immunohistochemistry

##### Cleaved Caspase-6 Staining

Immunohistochemical analysis was performed in both a human tissue sample and our animal studies to determine the protein expression of cleaved caspase-6 (cl-C6) or cleaved caspase-3 (cl-C3) at 8, 24 and 72 h after hypoxia-ischaemia and in controls. Immunohistochemical staining was performed as previously described [30] for the detection of cl-C6 using rabbit anti-cl-C6 (1:500; No. 9761; Cell Signaling, Hertfordshire, UK); for the cl-C3 reaction we used rabbit-anti-cl-C3(1:1,000; No. 9661L; Cell Signaling) and biotinylated goat anti-rabbit IgG secondary antibody (15 μg/ml; Vector Laboratories). As negative controls, we performed staining in the absence of the primary antibodies and pre-absorption with the recombinant caspase-6 (1 U; Enzo Life Sciences; Exeter, UK); no specific staining was identified in these preparations.

##### Immunofluorescence Double-Labelling

To identify the protein expression of cl-C6, double-labelling immunofluorescence was performed using a cocktail of rabbit anti-cl-C6 (1:500) with a monoclonal antibody specific for either astrocytes (GFAP, 1:1,000; Sigma-Aldrich) or oligodendrocytes (Olig2, 1:2,000; Millipore, Temecula, Calif., USA) and a secondary antibody cocktail consisting of goat anti-mouse IgG conjugated to Alexa Fluor 488 (4 μg/ml; Invitrogen, Eugene, Oreg., USA) and goat anti-rabbit IgG conjugated to Alexa Fluor 546 (4 μg/ml; Invitrogen). Immunofluorescence was performed as previously described [30].

For double-labelling with cl-C6 and the microglia we stained cl-C6 with Alexa Fluor 546. The slides were then immersed in 4% paraformaldehyde for 5 min and incubated with biotinylated *Lycopersicon esculentum* (tomato) lectin (1:1,000; Vector Laboratories) and followed with streptavidin-conjugated Alexa Fluor 488 (Vector Laboratories).

##### Image Acquisition

The total image was acquired using the CM1 and CM2 modules for virtual tissue scan (MicroBrightField, Inc., Colchester, Vt., USA). The sections were examined under bright-field microscopy using a light microscope (DM6000 B; Leica Microsystems Ltd., Milton Keynes, UK) equipped with a motorised specimen stage for automated sampling, CCD colour video camera and stereology software (Stereo Investigator, v8.27; MicroBrightField, Inc.). The average area of each contour was encompassed by an average area of 1.0 mm^2^ to provide a scanned image collected using a 40× objective (0.0426 mm^2^).

##### Brain Injury Assessment

Pups (caspase-6 WT, Het and KO) were killed and perfused intracardially with 0.9% physiological saline followed by 5% buffered formaldehyde (Histolab AB, Sweden). After dehydration through graded ethanol and xylene (X-TRA-Solv, Dalab, Sweden), brains were paraffin-embedded and serially cut into 7-μm coronal sections on Superfrost™ Plus slides (Thermo Scientific, Sweden) with every 50th section stained. To evaluate brain injury, sections were incubated with antibodies for microtubule-associated protein-2 (MAP-2), a neuronal marker expressed in neurons and dendrites, and myelin basic protein (MBP), a major constituent of the myelin sheath of oligodendrocytes where loss of staining indicated infarcted areas and MBP-associated white matter loss, respectively. Briefly, sections were dehydrated and underwent antigen retrieval with 0.01 m citric acid buffer (pH 6.0; 10 min heat, 20 min cool), peroxidase blocking with 3% H_2_O_2_ in PBS for 10 min, serum blocking with 4% horse serum, and overnight incubation at 4°C with either MAP-2 (1:1,000, Sigma-Aldrich, USA) or MBP (SMI-94, 1:10,000, Sternberger Monoclonal Inc., USA). Sections were then incubated for 1 h at room temperature with secondary antibody (horse anti-mouse, 1:250; Vector Laboratories, USA) followed by incubation for 1 h with avidin-biotin complex (Vector Laboratories, USA) and visualised with 3,3′-diaminobenzidine (DAB). Positive-stained areas were measured using the Olympus Micro Image analysis software system (V4.0, Olympus Optical, Tokyo, Japan).

Loss of MAP-2 and MBP staining was determined by subtracting the MAP-2- or MBP-positive area in the ipsilateral hemisphere from the contralateral hemisphere; values are expressed as a percentage of tissue loss from the contralateral hemisphere. Total volume loss was calculated according to the Cavalieri's principle: V = ΣA × P × T, where V = total volume, ΣA = sum of the areas, P = the inverse of the section's sampling fraction, and T = section thickness. MBP staining was measured in the subcortical white matter region at one level of the brain (approximately at the level of bregma −2.12 mm) [31].

#### Primary Microglial Cultures

Primary mixed glial cultures were prepared from P1 to P3 C57bl6 (WT) and caspase-6 KO mice of both sexes, as previously described [32], with some alterations. Briefly, brains were isolated and the cerebellum removed and washed in cold Hanks buffered salt solution (Sigma-Aldrich, USA) supplemented with 100 UI/ml penicillin and 100 μg/ml streptomycin (Sigma-Aldrich, USA). Forebrains were gently dissociated by titration in Dulbecco's modified Eagle's medium (DMEM; Sigma-Aldrich, USA) supplemented with 20% heat-inactivated fetal bovine serum (FBS; Gibco; Life Technologies Sweden) and 1% antibiotics. The cell suspension was passed through a 70-μm filter (Becton-Dickinson) and plated in 75-cm^2^ flasks with vented caps (Sarstedt, Germany) at a density of two brains per flask. After 7 days all the media was replaced with DMEM/10% FBS/antibiotics and cultured for a further 7 days. Microglia were then detached by shaking (250 rpm, for 3 h at 36°C) on a rotary shaker, and the microglia cell suspension was collected and centrifuged (250 *g* × 10 min). The media were then removed, the pellet was resuspended in DMEM/2% FBS/antibiotics and the number of cells were counted with an automated cell counter (Scepter; Millipore) and seeded into 12-well plates (approximately 2-2.5 × 10^5^ cells per well). Flasks and plates were incubated in a humidified oven at 37°C containing 5.1% CO_2_ in air.

#### In vitro Lipopolysaccharide and Q-VD-OPh Stimulation

After 24 h, the cell culture medium was replaced with DMEM/antibiotics. WT and caspase-6 KO primary microglial cells were stimulated with 10 ng/ml (diluted in sterile water) of *Escherichia coli* lipopolysaccharide (LPS; 055:B5; List Biological Laboratories, USA) or sterile water (control) - each 12-well plate always contained both treatments. We treated WT microglia with the broad-spectrum caspase inhibitor Q-VD-OPh (20 μm; diluted in DMSO; SM Biochemicals, USA) immediately prior to LPS stimulation. The dose of Q-VD-OPh was chosen based on the manufacturer's suggestions. Each 12-well plate in these experiments contained all treatments: control (DMSO + sterile water), LPS (DMSO + LPS 10 ng/ml), Q-VD-OPh only, and Q-VD-OPh + LPS. We also treated cells with negative control (Q-VE-OPh, 20 μm; SM Biochemicals, USA), which were also included on each plate (Q-VE-OPh only, Q-VE-OPh + LPS) - these data are not shown in this manuscript. The entire supernatant was collected (1 ml) at 6, 24 or 48 h; 300 μl was used immediately for a lactate dehydrogenase assay (Roche, USA) and the remaining supernatant stored at −80°C until further analysis. Cells were washed with RNAse-free PBS and then lysed with 350-μl buffer RLT (Qiagen; containing β-mercaptoethanol at a concentration of 1:100), harvested and stored at −80°C for gene expression analysis. All time points were run in each independent experiment and all group experiments were performed a minimum of 3-6 independent times.

#### Cytokine/Chemokine Assay

Cytokines and chemokines were measured in cell supernatants (50 μl) from primary microglia cultures using a Bio-Plex Pro mouse cytokine 23-plex assay (Bio-Rad, Sweden), following the manufacturer's protocol. Concentrations of IL-1α, IL-1β, IL-2, IL-3, IL-4, IL-5, IL-6, IL-9, IL-10, IL-12 (p40), IL-12 (p70), IL-13, IL-17a, eotaxin, granulocyte colony-stimulating factor (G-CSF), granulocyte macrophage colony-stimulating factor (GM-CSF), interferon-gamma (IFN-γ), KC/chemokine (C-X-C motif) ligand 1 (CXCL1), monocyte chemotactic protein-1 (MCP-1)/chemokine (C-C motif) ligand 2 (CCL2), macrophage inflammatory protein 1α (MIP-1α)/CCL3, MIP-1β/CCL4, RANTES, and TNF-α were simultaneously detected on a Bio Plex 200 System (Bio-Rad, Sweden). For some cytokines the values were too low or too high for accurate detection; if this occurred for 3 or more samples, no value was calculated.

### RT-qPCR

Total RNA from primary microglial cell were extracted with RNeasy micro kit (Qiagen, Sweden), following the manufacturer's instructions. RNA concentration and purity (260/280 ratio) were quantified spectrophotometrically with a NanoDrop (ND-1000 UV/Vis Spectrophotometer; Thermo Scientific, USA). A QuantiTect reverse transcription kit (Qiagen, Sweden) was used to synthesise first-strand cDNA from 75 ng of total RNA, following the manufacturer's instructions. RT-qPCR was performed in duplicate, with each PCR containing 2 μl cDNA, 10 μl QuantiFast SYBR Green PCR Kit (Qiagen, Sweden), 2 μl PCR Primer assay (Qiagen, Sweden), and 6 μl H_2_O.

The amplification protocol comprised an initial 5-min denaturation at 95°C followed by 45 cycles of denaturation at 95°C for 10 s and annealing/extension for 30 s at 60°C on a LightCycler 480 (Roche, Sweden). Melt curve analysis was performed to ensure that only one PCR product was obtained. A standard curve was also generated using increasing concentrations of cDNA, allowing for quantification and estimating amplification efficiency. Amplification transcripts were quantified with the relative standard curve and normalised against reference gene YWHAZ. Comparison of reference genes YWHAZ, GAPDH, GUSB, and 18S was done for all treatments, genotypes and time points using GenEx Software (v5.1.1.1; MultiD Analyses AB, Sweden), which uses GeNorm and NormFinder algorithms to calculate optimal reference genes. All primer details are listed in table 1.

### Data Analysis

Data are expressed as mean ± SEM. All primary microglia culture experiments were conducted a minimum of 3 independent times. All statistical analyses were performed using GraphPad Prism 6 Software (GraphPad Software, San Diego, Calif., USA), with significance set at p < 0.05. All data were assessed for normality. Brain injury analysis was conducted using a one-way ANOVA. Gene expression and multiplex measurements were assessed with a two-way ANOVA for treatment and time. If significant, a Dunnett's (comparing with control or WT LPS) or Sidak post hoc (more power than Bonferroni) analysis was conducted.

## Results

### Expression of cl-C6 in Human Preterm White Matter Injury

**Fig. 1:**
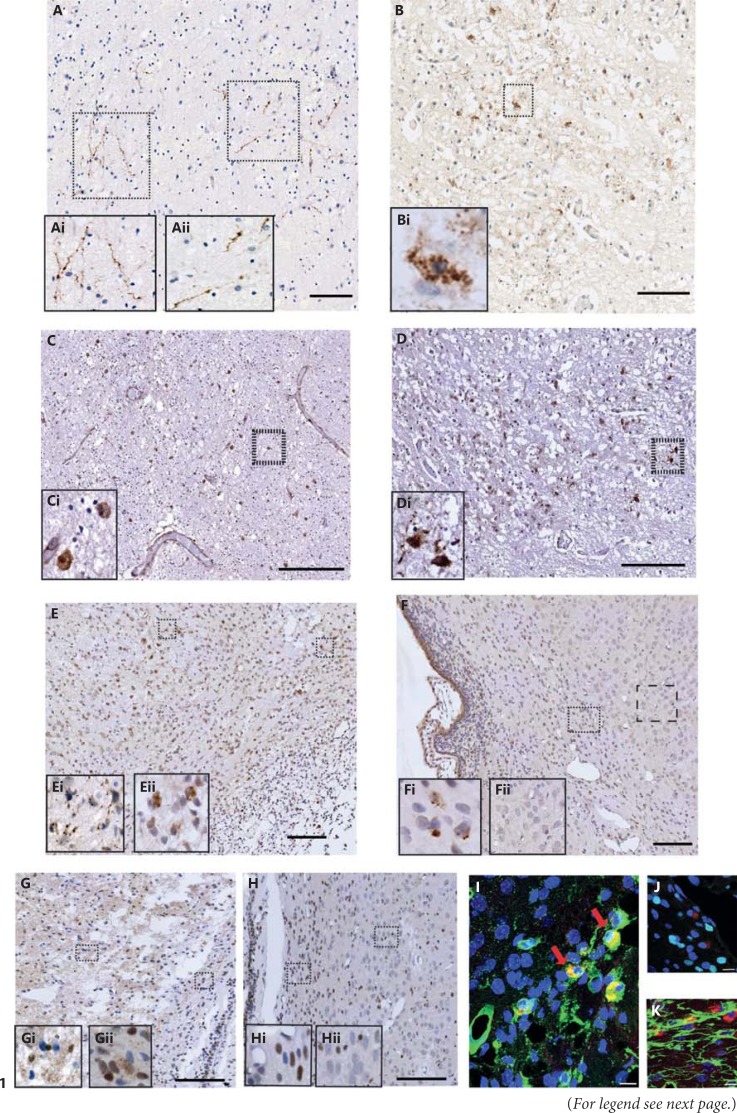
Cleaved caspases in the human preterm brain and in a neonatal hypoxic-ischaemic rat model. Immunoreactivity of cl-C6 (**A**, **B**) and cl-C3 (**C**, **D**) was expressed in a human preterm white matter injury case in the periventricular white matter (**A**, **C**) and anterior limb of the internal capsule (**B**, **D**). cl-C6 staining appeared fibrous (**Ai**, **Aii**) and perinuclear (**Bi**). Sporadic and nuclear cl-C3 was seen in the periventricular white matter region (**C**, **Ci**). However, the internal capsule the cl-C3 immunoreactivity was both nuclear and cytoplasmic (**D**). Following neonatal hypoxia-ischaemia in rats, cl-C6 (**E**, **F**) was observed in both the ipsilateral (**E**) and contralateral (**F**) hemispheres. Consistent with the observations made in the human tissue, cl-C6 again appeared to be fibrous in the cortex (**Ei**) and perinuclear in white matter (**Eii**) in the ipsilateral hemisphere, whereas in the contralateral side, there was perinuclear staining seen in the white matter (**Fi**) but not in the cortex (**Fii**). cl-C6 immunoreactivity was co-localised mainly in the microglia (tomato lectin; **G**) but not with oligodendroglia (Olig-2; **H**) or astroglia (GFAP; **I**). cl-C3 was also observed in rats following neonatal hypoxia-ischaemia, (**J**, **K**). Nuclear and punctate staining of cl-C3 was abundant in the ipsilateral side (**J**, **Ji**, **Jii**), but the contralateral side had primarily nuclear staining (**K**, **Ki**, **Kii**). Red arrows indicate co-labelled cells. Scale bar = 100 µm (**A-F**, **J**, **K**) and 10 µm (**G-I**).

In order to begin assessing the importance of caspase-6 activity in neonatal brain injury, we investigated whether cl-C6 could be seen in human post-mortem preterm brain sections where white matter injury had occurred. We identified cl-C6 immunostaining (a marker for caspase-6 activation) in fine fibrous (fig. 1Ai, Aii) and perinuclear cells (fig. 1Bi) in the periventricular white matter (fig. 1A) and anterior limb of the internal capsule (fig. 1B), respectively. Caspase-3 has been shown to be activated (cl-C3) within 24 h following hypoxia-ischaemia [33]. This post-mortem case had a post-menstrual age of 29.1 weeks (born at a gestational age of 24 weeks) and, in the same regions examined, showed regions of sporadic cl-C3-positive staining (fig. 1C, D).

### cl-C6 Is Expressed Following Neonatal Hypoxia-Ischaemia in the Rat Brain

To investigate the temporal expression and localisation of cl-C6, we used the well-characterised Vannucci model of hypoxic-ischaemic injury [28]. We performed immunohistochemistry for cl-C6 in control (P8) rat brain sections as well as sections taken at 8, 24 and 72 h following hypoxia-ischaemia. We detected sporadic cl-C6 immunostaining in white matter 72 h after hypoxia-ischaemia in both the ipsilateral (fig. 1E) and contralateral hemispheres (fig. 1F). In the cortex (fig. 1Ei), cl-C6 reactivity was detected in the damaged axonal processes on the ipsilateral side but not in axonal processes of the contralateral side.

#### Active Caspase-6 Was Identified in Microglia in the White Matter

To identify the cell types that expressed cl-C6 in white matter we utilised double-labelling immunofluorescence of cl-C6 antibody with glial markers for microglia (tomato lectin; fig. 1G), astroglia (GFAP; fig. 1H) and oligodendrocytes (Olig-2; fig. 1I). Double-label immunofluorescence revealed that cl-C6-positive cells were co-localised with microglia but not with astrocytes or oligodendrocytes. There is no single reported marker of activated microglia; therefore, we closely inspected the morphology of positively stained microglia. The somatic cl-C6 immunoreactivity that co-localised with the tomato lectin appeared to be present only in amoeboid-shaped cells, which is more indicative of activated microglia - interestingly, these cells did not appear to be of apoptotic morphology. We also observed Iba-1-positive microglia co-localising with cl-C6 at 72 h following hypoxia-ischaemia in WT mice (data not shown).

##### Cascade Response of Caspase-3 Expression Is Seen Following Neonatal Hypoxia-Ischaemia in the Rat Brain

cl-C3 is robustly upregulated in neonatal hypoxia-ischaemia [33] and is indicative of apoptotic changes occurring in the cell - identified by somatic blebbing or fragmentation. We compared the expression of cl-C6 with cl-C3 in the same tissue and found that ipsilateral hemisphere (fig. 1J) cortical regions were abundant with cl-C3 nuclear expression that extended into cell processes 72 h following hypoxia-ischaemia. This was particularly evident in the cortical region where punctate and fragmented staining was seen amongst the cl-C3-positive nuclei. Beneath the core of the infarcted area in the white matter region cl-C3 expression was both nuclear and fibrous. However, in the contralateral side (fig. 1K) the expression was in sporadic nuclei in both the cortical and white matter regions, but the fine punctate immunoreactivity in the processes was not present.

#### Neuropathology Outcome in Caspase-6 Knockout Mice Following Hypoxia-Ischaemia

Mixed litters were randomly subjected to either 50 min (WT, n = 16, 7 males, 9 females; Het, n = 25, 13 males, 12 females; KO, n = 22, 11 males, 11 females) or 60 min (WT, n = 18, 11 males, 7 females; Het, n = 26, 13 males, 13 females; KO, n = 18, 10 males, 8 females) of hypoxia-ischaemia.

##### Brain Injury Evaluation

**Fig. 2:**
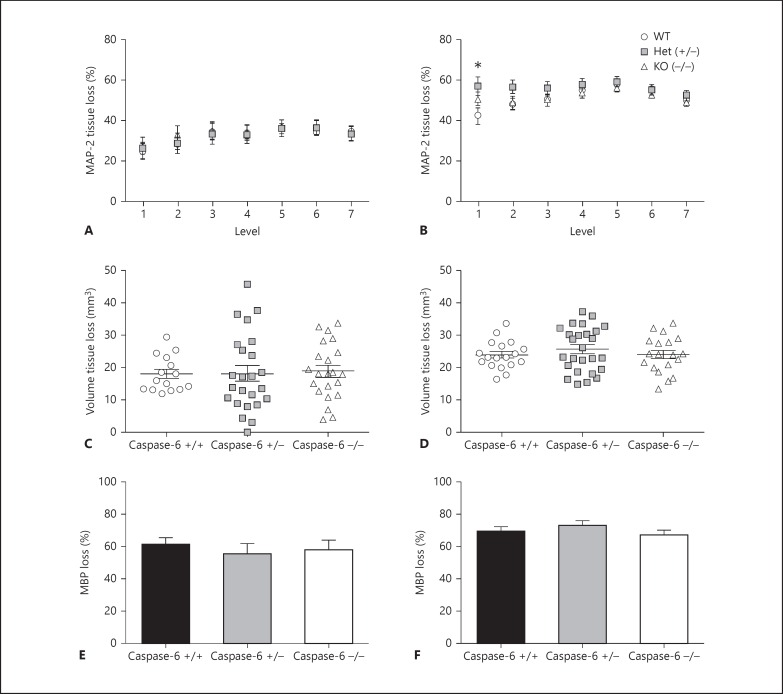
Neuropathology assessment of caspase-6 WT, Het and KO mice 72 h following 50 or 60 min of hypoxia-ischaemia at P9. Tissue loss (**A**, **B**) was assessed using MAP-2 staining at 7 levels (posterior to anterior) of the brain in caspase-6 +/+ (WT; open black circles), caspase-6 +/- (Het; grey squares) and caspase-6 -/- (KO; open black triangles). Volume tissue loss assessment (**C**, **D**) and subcortical white matter loss at the level of the hippocampus (**E**, **F**) showed no significant difference between genotypes following 50 min (**A**, **C**, **E**) or 60 min (**B**, **D**, **F**) of hypoxia-ischaemia (H-I). Mean ± SEM. * p < 0.05

Brain injury was assessed 72 h following 50 or 60 min of hypoxia-ischaemia in 7 levels of the brain (level 1 corresponding to the posterior brain). There was no significant difference between genotypes (WT, Het or KO) following 50 min of hypoxia-ischaemia (fig. 2A). At the posterior level of the brain (level 1) there was a significant (p = 0.05) difference between genotypes, where Het mice showed greater injury (57.12 ± 4.59%) compared with WT (42.52 ± 4.01%), but there was no significant difference at any other levels analysed (fig. 2B). There was also no significant difference in the volume of tissue loss following 50 min (fig. 2C) or 60 min (fig. 2D) of hypoxia-ischaemia.

##### Subcortical White Matter Loss

Injury to the subcortical white matter at the level of the hippocampus was assessed using MBP immunohistochemistry. Gene deletion of caspase-6 did not have any significant effect on loss of white matter following 50 min (fig. 2E) or 60 min (fig. 2F) of hypoxia-ischaemia.

#### Caspase Expression in Primary Microglia

##### Alterations in Caspase Gene Expression in Primary Microglia Following LPS Stimulation

**Fig. 3:**
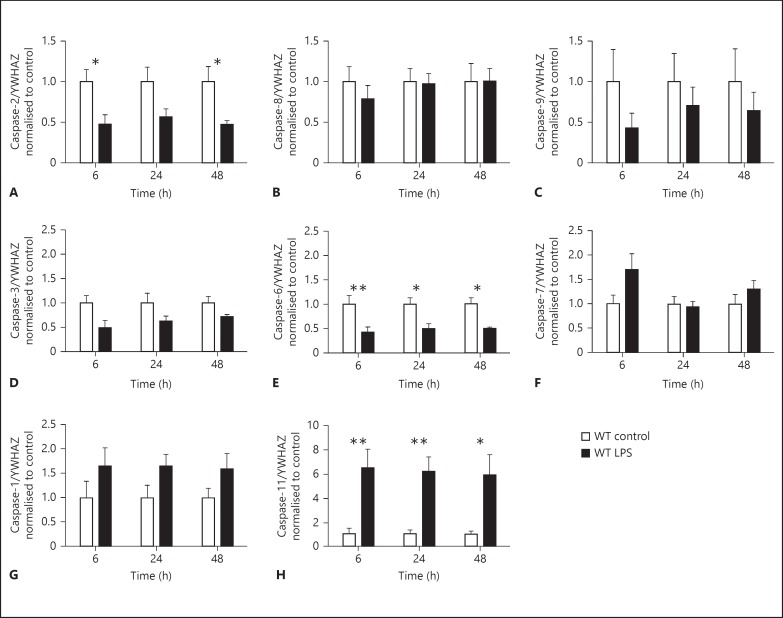
Caspase mRNA in primary microglia following LPS (10 ng/ml). Gene expression for the following caspases was assessed in WT primary microglia: initiator - caspase-2 (**A**), caspase-8 (**B**) and caspase-9 (**C**); effector - caspase-3 (**D**), caspase-6 (**E**) and caspase-7 (**F**), and inflammatory - caspase-1 (**G**) and caspase-11 (**H**). LPS-treated microglia (black bars) were normalised and compared with control (white bars) cells for each time point. Data were analysed with a two-way ANOVA (for time and treatment) and, when significant, a Sidak post hoc analysis was conducted. Data are mean ± SEM (n = 5 independent experiments). * p < 0.05, ** p < 0.01.

As our previous results suggested that caspase-6 may play a role in activated microglia, we assessed which caspases were present in primary microglia and determined whether stimulation with low-dose LPS (10 ng/ml) would modulate their expression. We found microglial expression of all caspases measured and significant treatment effects were seen for mRNA expression of caspase-2 (fig. 3A, p < 0.001), caspase-3 (fig. 3D, p < 0.01), caspase-6 (fig. 3E, p < 0.0001), caspase-1 (fig. 3G, p < 0.05), and caspase-11 (fig. 3H, p < 0.0001). Post hoc analysis revealed that mRNA expression of caspase-2 (an initiator caspase) and caspase-6 (an effector caspase) was significantly downregulated following LPS stimulation. In contrast, the expression of caspase-11 (an inflammatory caspase) was upregulated at 6, 24 and 48 h following LPS stimulation. No differences were seen in caspase-8 (fig. 3B), caspase-9 (fig. 3C) or caspase-7 (fig. 3F).

To check the efficacy of the pan-caspase inhibitor Q-VD-OPh (20 µm) and/or LPS (10 ng/ml) on caspase activity in primary microglia we used Caspase-Glo® (Promega, USA; as per manufacturer's instructions on 4 independent experiments) substrates 3/7 (Z-DEVD), 6 (Z-VEID) and 8 (Z-LETD) at 6, 24 and 48 h after treatment. Caspase activity was not influenced by low-dose administration of the pro-inflammatory TLR-4 agonist LPS. Caspase-3/7 (by 95%), caspase-8 (by 92%) and caspase-6 (by 57%) were all substantially reduced at 48 h after treatment with the broad-spectrum inhibitor Q-VD-OPh (data not shown). The data demonstrate that the dose of Q-VD-OPh was sufficient to reduce microglial caspase activity, but the relative contribution of the different caspases remains unclear because of the overlap in specificity of the substrates used [34,35,36]. We also conducted lactate dehydrogenase measurements, an assessment of cell membrane integrity, on cell supernatants and found no difference across time or between treatment groups (data not shown), suggesting that the dose of LPS and the administration of Q-VD-OPh had no effect on microglial viability.

#### Cytokine and Chemokine Expression Following Microglial Stimulation with Q-VD-OPh and Caspase-6 KO

To determine the effect of caspase-6 activity on microglial activation and phenotype we prepared primary microglial cultures from caspase-6 KO mice and measured cytokines and phenotypic markers. As caspase-3/7 and caspase-8 signalling has been reported to mediate microglial activation [24], we compared the caspase-6 KO microglia with WT microglia treated with Q-VD-OPh.

#### Microglial Activation

**Fig. 4:**
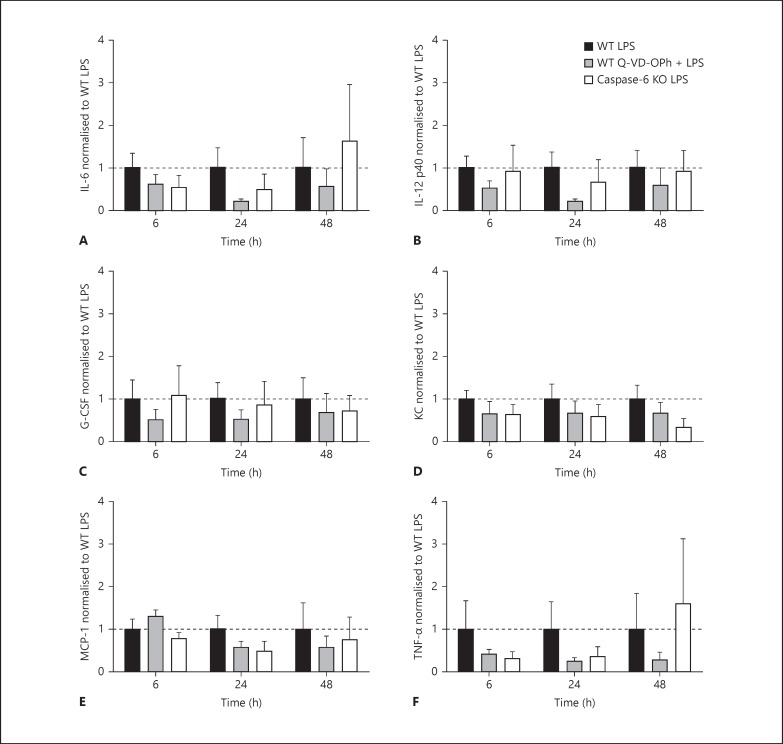
Cytokine expression from microglial cell supernatants. IL-6 (**A**), IL-12 p40 (**B**), G-CSF (**C**), KC (**D**), MCP-1 (**E**) and TNF-α (**F**) measured from microglial cell supernatants at 6, 24 and 48 h. Data are expressed as a fold change from WT LPS treatment for each time point. Data were analysed with a two-ANOVA for time and treatment. Data are mean ± SEM (n = 5-6 independent experiments). Dotted line indicates 1.

Cell supernatants were analysed using a 23-plex mouse cytokine panel. No differences were observed between WT control, WT Q-VD-OPh and caspase-6 KO control groups (table 2). LPS treatment resulted in increased expression of most of the cytokines analysed: IL-1α, IL-1β, IL-2, IL-3, IL-4, IL-5, IL-9, IL-10, IL-12p70, IL-13, IL-17, CCL11 (eotaxin), GM-CSF, IFN-γ, CCL3 (MIP-1α), CCL4 (MIP-1β), CCL2 (MCP-1) and CCL5 (RANTES; table 2). Inhibition of caspase activity by Q-VD-OPh or deletion of the caspase-6 gene in microglia did not significantly influence the LPS-induced changes of IL-6, IL-12p40, G-CSF, KC, MCP-1, and TNF-α (fig. 4).

#### Microglial Phenotype

**Fig. 5:**
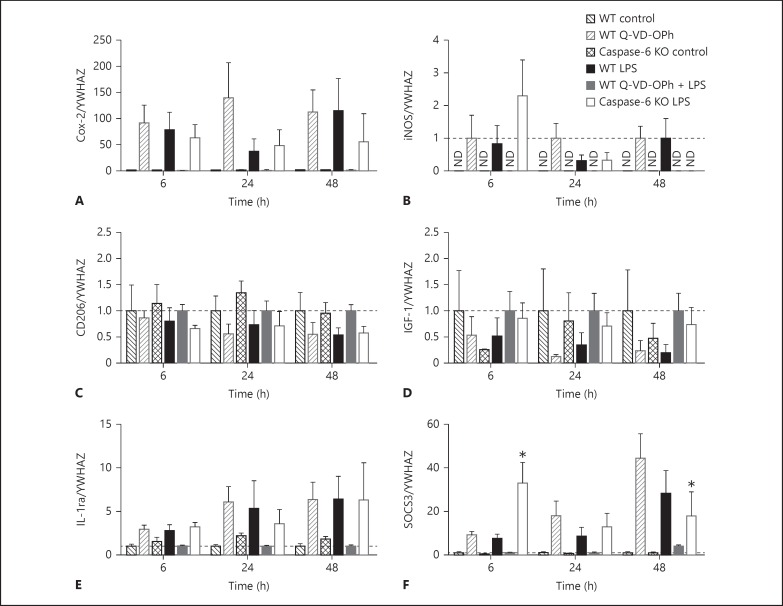
Gene expression of microglial phenotype markers in WT primary microglia following LPS (10 ng/ml), Q-VD-OPh (20 µm), Q-VD-OPh + LPS, and caspase-6 KO control and LPS. Expression of genes indicative of M1: Cox-2 (**A**) and iNOS (**B**); M2a: CD206 (**C**) and IGF-1 (**D**), and M2b: IL-1ra (**E**) and SOCS3 (**F**), was normalised to WT control (black checked bars) for WT LPS (black bars), WT Q-VD-OPh (dark grey checked bars), WT Q-VD-OPh + LPS (dark grey bars) and caspase-6 KO LPS (white bars) was normalised to caspase-6 KO control (checked bars). For iNOS (**B**) gene expression, only LPS-treated cells were detected, so data were normalised to WT LPS. Data were analysed with a two-way ANOVA (for time and treatment) and, when significant, a Dunnett's post hoc analysis was conducted comparing with WT LPS treatment. ND = Not detected. Data are mean ± SEM (n = 4-5 independent experiments; iNOS: n = 3-4 independent experiments). Dotted line indicates 1. * p < 0.05.

In a previous study, Chhor et al. [37] detailed microglial phenotype markers, of which we chose six representing M1 (classical) cytotoxic (COX-2 and iNOS; fig. 5A, B), M2a tissue repair (CD206 and IGF-1; fig. 5C, D) and M2b immunosuppressive (IL-1ra and SOCS3; fig. 5E, F) phenotypes. Gene expression of microglial phenotype markers was assessed in LPS-treated caspase-6 KO microglia or WT microglia ± Q-VD-OPh at 6, 24 and 48 h (fig. 5). Interestingly, SOCS3 gene expression (fig. 5F) showed a significant time (p < 0.05), treatment (p < 0.0001) and interaction (p < 0.01) effect. Post hoc analysis also revealed that caspase-6 KO microglia stimulated with LPS showed a different temporal expression compared with LPS-treated WT cells, peaking significantly earlier at 6 h and being significantly lower than WT LPS treatment at 48 h. For WT control, WT Q-VD-OPh and caspase-6 KO control cells, gene expression for iNOS was not detected; therefore, data were normalised to WT LPS treatment for each time point.

## Discussion

In the present study we examined the expression and possible role of caspase-6 in the immature brain after hypoxia-ischaemia and in primary microglia exposed to LPS. cl-C6 was present but expressed at a low level in a human post-mortem case of preterm white matter injury. In a rat model of hypoxia-ischaemia, caspase-6 expression was low and primarily evident in white matter at later time points compared with cl-C3, which is robustly expressed in the immature brain. Caspase-6 gene deletion did not affect the extent of brain injury following mild (50 min) or severe (60 min) hypoxia-ischaemia. However, we found that cl-C6 was present predominantly in activated microglia at 72 h following hypoxia-ischaemia. Previous studies have shown that administration of a broad-spectrum caspase inhibitor, Q-VD-OPh, offers neuroprotection following hypoxia-ischaemia and, therefore, we explored possible deleterious functions of caspases in primary microglia. We report subtle changes in the activation state of caspase-6 and other caspase family members and alterations in the production of cytokines in microglia following low-dose LPS stimulation.

The cellular expression of cl-C6 observed by us in the human preterm brain in the presence of white matter injury may highlight a subtle role for caspase-6 in the development of secondary [38] and tertiary brain injury [39]. Caspase-6 is traditionally described as an effector caspase and has recently been implicated in the process of axonal degeneration during neuronal apoptosis in ageing [14,40]. The activation of caspase-6 has been shown to be regulated by caspase-1 [41], caspase-3/7 [14,42] and caspase-9 [19], and caspase-6 itself can undergo self-activation [43]. Caspase-6 can also activate caspase-3 and caspase-8 [13,44,45], suggesting a more complicated function than first detailed.

Consistent with our findings in the human tissue, we also observed cl-C6 staining at 72 h following hypoxia-ischaemia in cell bodies and cell processes of white matter in the immature brain. This is in contrast with the adult brain following ischaemia-reperfusion (medial cerebral artery occlusion), where cl-C6 was present from 12 to 72 h in the cell body and processes of neurons [19]. Whilst the models of ischaemia result in different injury patterns, the delayed activation of caspase-6 seen in the immature brain may be representative of both low pro-caspase-6 expression seen in fetal human tissue [15] and high caspase-3 expression. It has been reported that caspase-3 is required to activate caspase-6 [14], and we propose that the presence of cl-C6 at 72 h following hypoxia-ischaemia (fig. 1) may be the downstream effect of capase-3 activity which peaks at 24 h following hypoxia-ischaemia [33]. We did attempt to optimise and assess capase-6 activity (substrate Ac-Val-Glu-IIe-Asp-AMC) in brain homogenates following hypoxia-ischaemia but were unsuccessful. Cleavage of lamin A/C has also been shown to be a specific indicator of caspase-6 activity [46], but we were unable to detect it in our samples as lamin A/C expression is only present in a minority of cells at P5 and P10 and in most cells at P15 in the mouse [47].

Our findings strongly indicate that in the immature brain caspase-3 is the most significant effector caspase - important in both brain development and apoptosis following hypoxia-ischaemia. This is in comparison with caspase-6, which appears to be an ‘age-dependent’ caspase, increasing expression and function during ageing [18]. This is further strengthened by our result that genetic deletion of caspase-6 was not protective in our neonatal model of hypoxia-ischaemia. It has been reported that neurons from caspase-6 knockouts are protected from excitotoxic injury, and as caspase-6 knockout mice age they have larger cortical and striatal volumes and develop learning deficits [48]. Akpan et al. [19] also found that caspase-6 knockout mice were protected from transient middle cerebral artery occlusion, retaining more neurons. However, all of these assessments have been performed in mice from the age of 2 months and onwards. Therefore, the functional role of cl-C6 remains to be elucidated in the immature brain. The fibrous staining we observed in the preterm brain and 72 h following hypoxia-ischaemia could indicate the initiation of fine axonal pruning and degeneration. Cusack et al. [49] have recently found that caspase-6-dependent and -independent pathways degenerate axons, and this is reliant on whether cells have experienced axon-specific (pruning) or whole cell apoptosis. This finding remains to be investigated further in our tissue by assessing later time points. Importantly, we observed cl-C6 co-localisation with amoeboid microglia in white matter following hypoxia-ischaemia. These cells were not of apoptotic morphology, and hence we decided to investigate the possible non-apoptotic role of caspase-6 in LPS-stimulated primary microglia. Microglia are the resident phagocytic cells of the brain that are required during brain development and following tissue injury. Their involvement during neuronal apoptosis may suggest an interaction between apoptotic cues such as caspase activation and microglial activation.

We showed that the mRNA of a number of caspases was modulated following low-dose LPS stimulation (10 ng/ml), but caspase activity (using LETD, DEVD, VEID substrates) was not affected by LPS. Our findings are consistent with Kobayashi et al. [50], who also found that LPS did not induce or require caspase-6 activity in THP-1 macrophages. Burguillos et al. [24] have shown that caspase-3/7 and caspase-8 signalling can mediate microglial activation. Therefore, for comparison with caspase-6 knockout microglia, we also treated cells with Q-VD-OPh, a broad-spectrum caspase inhibitor that has been shown to be neuroprotective in neonatal models of hypoxia-ischaemia [9,10,11]. Q-VD-OPh targets caspases-1, −2, −3, −6, −8, −9, and −10. It effectively prevents apoptosis and, importantly, has a high potency at low concentrations and is non-toxic even at high concentrations [51,52]. Whilst neuroprotective properties of Q-VD-OPh are primarily based on its anti-apoptotic function, the possible non-apoptotic effect Q-VD-OPh has on cell types such as microglia has not be investigated.

Microglia have both beneficial and detrimental actions. To better understand their function, the description of microglia phenotype or activation states has been sub-classified in classical activation (M1: pro-inflammatory cytokine production), alternate activation (M2a: anti-inflammatory cytokine production) and acquired deactivation (M2b - immunosuppressive). Chhor et al. [37] have described temporal changes in phenotype markers, from which we chose six markers that best represent each activation state. We found that caspase-6 knockout microglia displayed a different temporal expression of the M2b marker SOCS3 (suppressor of cytokine signalling), with an earlier maximum expression at 6 h compared with WT LPS microglia where SOCS3 expression peaked at 48 h following stimulation. The possible impact this may have on the microglia response remains to be elucidated. The M1 or M2a microglial phenotype markers were not significantly affected by caspase-6 gene deletion or by Q-VD-OPh.

Q-VD-OPh and caspase-6 gene deletion did not affect the production of cytokines in activated microglia. This is in contrast to Burguillos et al. [24], where caspase-3 and caspase-8 inhibition administered 1 h prior to high-dose LPS (100 ng/ml) resulted in a reduction of cytokines (IL-1β, IL-2, IL-4, IL-5, IL-10, IL-12, IFN-γ). Caspase-6 knockout mice are reported to have a reduction in adult pulmonary macrophage production of TNF-α [50], but we did not observe this in our caspase-6 knockout primary microglial preparations. Interestingly, microglial cells treated with recombinant caspase-6 enhanced the production of TNF-α [25] and regulated synaptic plasticity and inflammatory pain, suggesting an interaction between caspase-6 and TNF-α that requires further investigation. Whilst the differences we observed following caspase inhibition and caspase-6 gene deletion were subtle, methodological considerations such as time of inhibitor administration, higher doses of LPS stimulation and choice of different stimulants which can polarise microglia into different phenotypes may yield different results.

In summary, we found that the contribution of caspase-6 to the development of acute injury in neonatal hypoxia-ischaemia is subtle and likely to be predominantly regulated by caspase-3 as well as upstream caspase-9, −2 and −8 [2,3,4,6,8,12]. However, we found unique temporal regulation of caspase-6 activation in microglia, suggesting that caspase-6 may have a role in subsequent injury progression. Further studies are required in order to determine the role of caspase-6 at later time points.

## Figures and Tables

**Table 1:** Primers used for qPCR

Gene	NCBI ID	EntrezGene ID	Primer	QuantiTect primer set	Amplified exons	Amplicon length, bp
YWHAZ	NM_001253805 (3,604 bp) NM_001253806 (3,329 bp) NM_001253807 (3,160 bp) NM_011740 (3,801 bp)	22631	Mm_Ywhaz_1_SG	QT00105350	3/4	99

*Caspases*						
Caspase-1	NM_009807 (1,533 bp)	12362	Mm_Casp1_1_SG	QT00199458	6/7	101
Caspase-2	NM_007610 (3,463 bp)	12366	Mm_Casp2_1_SG	QT00108745	1/2	81
Caspase-3	NM_009810 (1,466 bp)	12367	Mm_Casp3_2_SG	QT01164779	3/4/5	150
Caspase-4	NM_007609 (1,433 bp)	12363	Mm_Casp4_1_SG	QT00096117	6/7	81
Caspase-6	NM_009811 (1,455 bp)	12368	Mm_Casp6_2_SG	QT00494921	6/7	125
Caspase-7	NM_007611 (2,350 bp)	12369	Mm_Casp7_1_SG	QT01058085	2/3	135
Caspase-8	NM_001080126 (2,412 bp) NM_009812 (2,585 bp) NM_001277926 (2,528 bp)	12370	Mm_Casp8_1_SG	QT00171437	7/8	74
Caspase-9	NM_015733 (3,896 bp)	12371	Mm_Casp9_1_SG	QT00133280	5/6/7	189

*Microglial phenotype*						
COX2 (Ptgs)	NM_011198 (4,150 bp)	19225	Mm_Ptgs2_1_SG	QT00165347	5/6	95
iNOS (NOS2)	NM_010927 (3,990 bp)	18126	Mm_Nos2_va.1_SG	QT01535800	3/4	131
IGF-1	NM_001111274 (7,039 bp) NM_010512 (7,121 bp)	16000	Mm_Igf1_1_SG	QT00154469	3/4	114
CD206 (MRC)	NM_008625 (5,322 bp)	17533	Mm_Mrc1_1_SG	QT00103012	28/29/30	132
SOCS3	NM_007707 (2,742 bp)	12702	Mm_Socs3_3_SG	QT02488990		90
IL-1ra	NM_001039701 (2,480 bp) NM_001159562 (2,409 bp) NM_031167 (2,464 bp)	16181	Mm_Il1rn_1_SG	QT00096054	2/3/4	116

**Table 2:** Multiplex analysis of microglial cell supernatants

Analyte	Treatment	6 h	24 h	48 h
IL-1α	Control	0.73±0.21	1.680.61	2.13±0.65
	LPS	3.79±0.84	9.14±1.79	23.03±12.74**
	Q-VD-OPh	0.82±0.31	2.25±0.64	2.57±0.81
	Q-VD-OPh + LPS	3.46±0.65	6.34±0.52	15.87±5.38
	Caspase-6 KO control	0.30±0.05	1.19±0.25	1.31±0.22
	Caspase-6 KO LPS	2.44±0.44	5.28±0.81	15.28±7.12**

IL-1β	Control	10.27±2.35	36.66±10.08	51.40±10.28
	LPS	108.91±18.43***	214.48±15.81****	255.88±21.37****
	Q-VD-OPh	19.02±5.53	57.40±15.02	70.89±10.94
	Q-VD-OPh + LPS	111.49±13.12***	195.42±20.04****	229.66±27.64****
	Caspase-6 KO control	ND<	16.48±4.48	24.57±6.84
	Caspase-6 KO LPS	70.70±22.13	145.15±44.89*	195.92±39.78**

IL-2	Control	ND<	ND<	ND<
	LPS	3.72±1.51	5.86±1.33	6.63±1.77
	Q-VD-OPh	ND<	ND<	2.11±0.92
	Q-VD-OPh + LPS	3.90±1.63	5.21±1.53	7.15±2.23
	Caspase-6 KO control	ND<	ND<	ND<
	Caspase-6 KO LPS	1.96±0.53	ND<	4.56±1.61

IL-3	Control	0.33±0.08	0.57±0.13	1.01±0.13
	LPS	1.66±0.41	4.27±1.19***	5.06±0.99***
	Q-VD-OPh	0.26±0.04	0.97±0.16	1.26±0.17
	Q-VD-OPh + LPS	1.75±0.38	3.18±0.70*	4.14±0.86**
	Caspase-6 KO control	0.15±0.03	0.43±0.17	0.54±0.17
	Caspase-6 KO LPS	1.13±0.43	2.46±0.90	4.92±1.84**

IL-4	Control	0.70±0.19	1.49±0.50	1.93±0.60
	LPS	3.24±0.93	7.45±2.06***	8.70±1.74***
	Q-VD-OPh	0.96±0.29	1.99±0.60	2.51±0.83
	Q-VD-OPh + LPS	2.97±0.61	5.81±1.15*	7.93±1.60***
	Caspase-6 KO control	0.69±0.05	1.43±0.21	1.65±0.17
	Caspase-6 KO LPS	2.64±0.48	5.24±0.67*	8.21±2.14***

IL-5	Control	0.10±0.02	0.22±0.06	0.33±0.09
	LPS	0.61±0.11	1.68±0.36****	1.87±0.35****
	Q-VD-OPh	0.24±0.07	0.34±0.07	0.41±0.08
	Q-VD-OPh + LPS	0.53±0.16	1.17±0.23**	1.57±0.27***
	Caspase-6 KO control	ND<	ND<	0.32±0.07
	Caspase-6 KO LPS	0.56±0.16	1.34±0.43	1.89±0.54*

IL-9	Control	ND<	ND<	ND<
	LPS	41.10±9.15	71.43±15.68	83.51±10.33
	Q-VD-OPh	ND<	17.58±3.22	15.33±3.45
	Q-VD-OPh + LPS	34.44±3.69	54.00±4.33	70.77±6.21
	Caspase-6 KO control	ND<	ND<	ND<
	Caspase-6 KO LPS	ND<	ND<	ND<

IL-10	Control	1.73±0.51	4.62±1.52	8.08±1.44
	LPS	14.83±2.68*	32.91±4.05****	44.13±5.43****
	Q-VD-OPh	2.33±0.78	7.30±1.08	8.90±2.21
	Q-VD-OPh + LPS	14.63±2.29*	29.94±4.90****	39.27±4.84****
	Caspase-6 KO control	0.82±0.29	3.57±0.81	5.39±1.41
	Caspase-6 KO LPS	11.19±3.55	24.38±9.03*	33.13±8.72**

IL-12p70	Control	2.62±0.52	15.25±3.85	16.95±5.72
	LPS	37.28±6.98	102.00±19.80****	113.84±15.63****
	Q-VD-OPh	11.91±4.15	16.44±4.68	23.56±8.44
	Q-VD-OPh + LPS	37.29±5.36	76.69±7.19***	101.98±7.91****
	Caspase-6 KO control	ND<	ND<	5.37±1.31
	Caspase-6 KO LPS	20.02±5.65	54.23±15.19	105.16±42.36

IL-13	Control	ND<	29.84±8.02	34.92±8.16
	LPS	97.17±31.48	173.48±40.41*	232.22±50.95***
	Q-VD-OPh	ND<	51.07±19.78	57.49±12.22
	Q-VD-OPh + LPS	88.34±20.85	154.10±30.59*	213.85±61.14**
	Caspase-6 KO control	ND<	21.88±3.11	27.84±5.86
	Caspase-6 KO LPS	53.61±13.21	99.12±29.34	129.46±28.71**

IL-17	Control	0.26±0.05	0.83±0.19	1.03±0.25
	LPS	2.02±0.35	4.98±0.81****	4.96±1.03****
	Q-VD-OPh	0.48±0.23	0.99±0.17	1.67±0.55
	Q-VD-OPh + LPS	1.95±0.24	3.81±0.24**	4.16±0.85***
	Caspase-6 KO control	ND<	ND<	0.52±0.26
	Caspase-6 KO LPS	1.58±0.41	2.73±0.75	4.49±1.54

Eotaxin	Control	ND<	84.41±14.12	105.22±30.39
(CCL11)	LPS	291.37±52.82	746.71±93.30**	1,012.50±163.60****
	Q-VD-OPh	ND<	153.74±29.38	183.37±61.77
	Q-VD-OPh + LPS	278.22±56.34	649.69±137.88**	857.23±171.55***
	Caspase-6 KO control	ND<	ND<	ND<
	Caspase-6 KO LPS	216.27±89.28	512.86±225.40	675.32±217.59

GM-CSF	Control	ND<	ND<	ND<
	LPS	63.67±22.04	117.84±35.65	141.13±32.00
	Q-VD-OPh	ND<	ND<	ND<
	Q-VD-OPh + LPS	72.82±18.12	100.06±25.17	123.76±29.88
	Caspase-6 KO control	ND<	44.52±8.49	44.56±9.57
	Caspase-6 KO LPS	57.53±12.27	98.93±10.39	146.41±42.58

IFN-γ	Control	ND<	3.09±1.18	2.07±0.80
	LPS	7.94±3.45	16.70±4.43	21.18±4.85**
	Q-VD-OPh	ND<	5.77±2.53	3.80±1.26
	Q-VD-OPh + LPS	6.11±1.80	13.71±3.08	19.21±5.80**
	Caspase-6 KO control	ND<	ND<	ND<
	Caspase-6 KO LPS	3.00±0.69	6.88±2.22	12.47±4.50

MIP-1α	Control	35.55±13.07	230.49±89.53	367.82±138.60
	LPS	1,440.85±451.02	6,521.97±867.18	>ND
	Q-VD-OPh	78.39±31.86	373.96±98.12	738.50±260.24
	Q-VD-OPh + LPS	1,033.63±153.93	5,705.81±949.03	>ND
	Caspase-6 KO control	42.06±3.61	133.82±20.33	209.50±39.70
	Caspase-6 KO LPS	697.56±265.09	2,065.97±956.10*	3,307.88±1,301.69**

MIP-1β	Control	73.77±33.28	738.32±312.24	1,358.61±572.17
	LPS	1,583.73±478.34	10,558.11±6,792.96*	8,525.95±2,119.06
	Q-VD-OPh	137.76±67.86	1,091.05±440.80	3,351.81±1,461.50
	Q-VD-OPh + LPS	1,178.20±234.30	5,843.26±1,764.86	8,970.78±3,321.53
	Caspase-6 KO control	88.48±22.47	527.99±128.50	1,178.52±365.24
	Caspase-6 KO LPS	1,140.15±421.87	4,278.15±2,320.81	7,043.62±2,438.00*

RANTES	Control	0.21±0.12	1.27±0.73	1.93±0.58
	LPS	18.72±7.14	467.31±143.26	2,350.72±861.91****
	Q-VD-OPh	0.19±0.10	1.79±0.49	12.89±5.92
	Q-VD-OPh + LPS	9.99±3.79	324.71±85.46	1,490.92±568.52***
	Caspase-6 KO control	0.27±0.08	1.20±0.31	2.47±0.27
	Caspase-6 KO LPS	13.68±7.04	784.24±599.44	1,347.62±1,042.26

Cytokine and chemokine production (pg/ml) analysed from microglia cell supernatants collected at 6, 24 and 48 h from WT control, WT LPS, WT Q-VD-OPh, WT Q-VD-OPh + LPS, caspase-6 KO control, and caspase-6 KO LPS treated cells. Data were analysed with a two-way ANOVA (for time and treatment) and, when significant, a Dunnett's post hoc analysis was conducted comparing WT control with WT LPS, WT Q-VD-OPh and WT Q-VD-OPh + LPS, or a Sidak post hoc analysis was carried out comparing caspase-6 KO control and caspase-6 KO LPS. ND<: values were below detectable range, >ND: values were above detectable range. Data are expressed as means ± SEM (n = minimum of 5 independent experiments).

* p < 0.05,

** p < 0.01,

*** p < 0.001,

**** p < 0.0001.
